# Supplementary material for: Narciclasine as a Novel Treatment for Lung Cancer and Malignant Pleural Mesothelioma: Insights from 3D Tumor Spheroid Models
Source: Int J Mol Sci. 2025 Oct 17;26(20):10127. doi: 10.3390/ijms262010127 (PMC12564550; doi:10.3390/ijms262010127)

Figure S1. Effects of Narciclasine on the size of LUAD and MM spheroids.

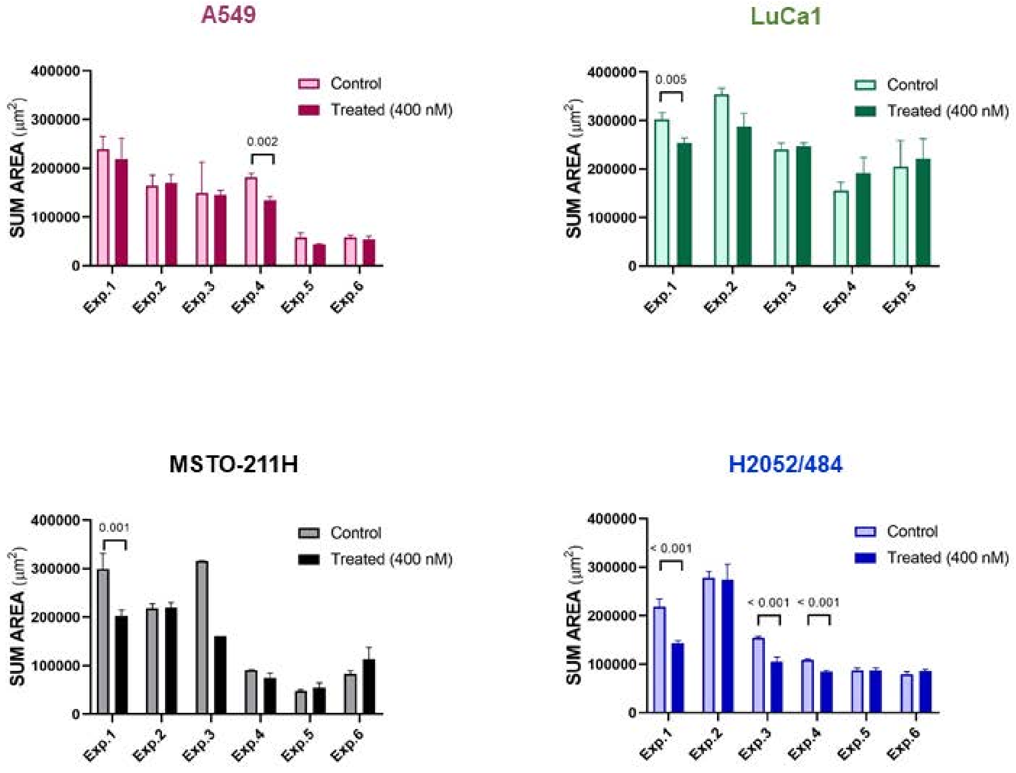

Figure S2. LDH activity in the negative (DMSO 0.2%) and positive control (Triton X-100 0.1%) conditions.

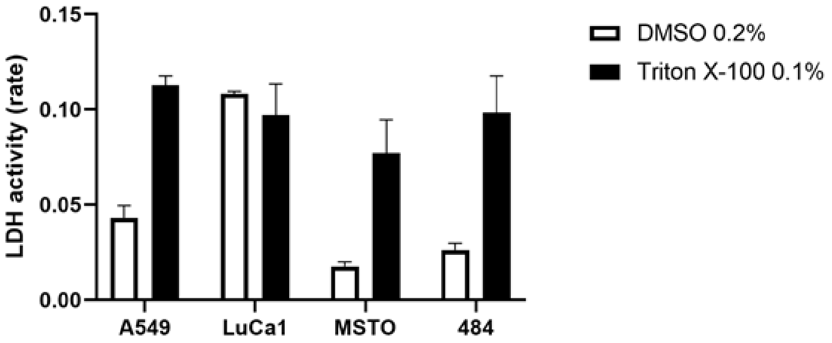

Figure S3. (**A-D**) Effects of Narciclasine on the proliferation and apoptosis of LUAD and MM spheroids.

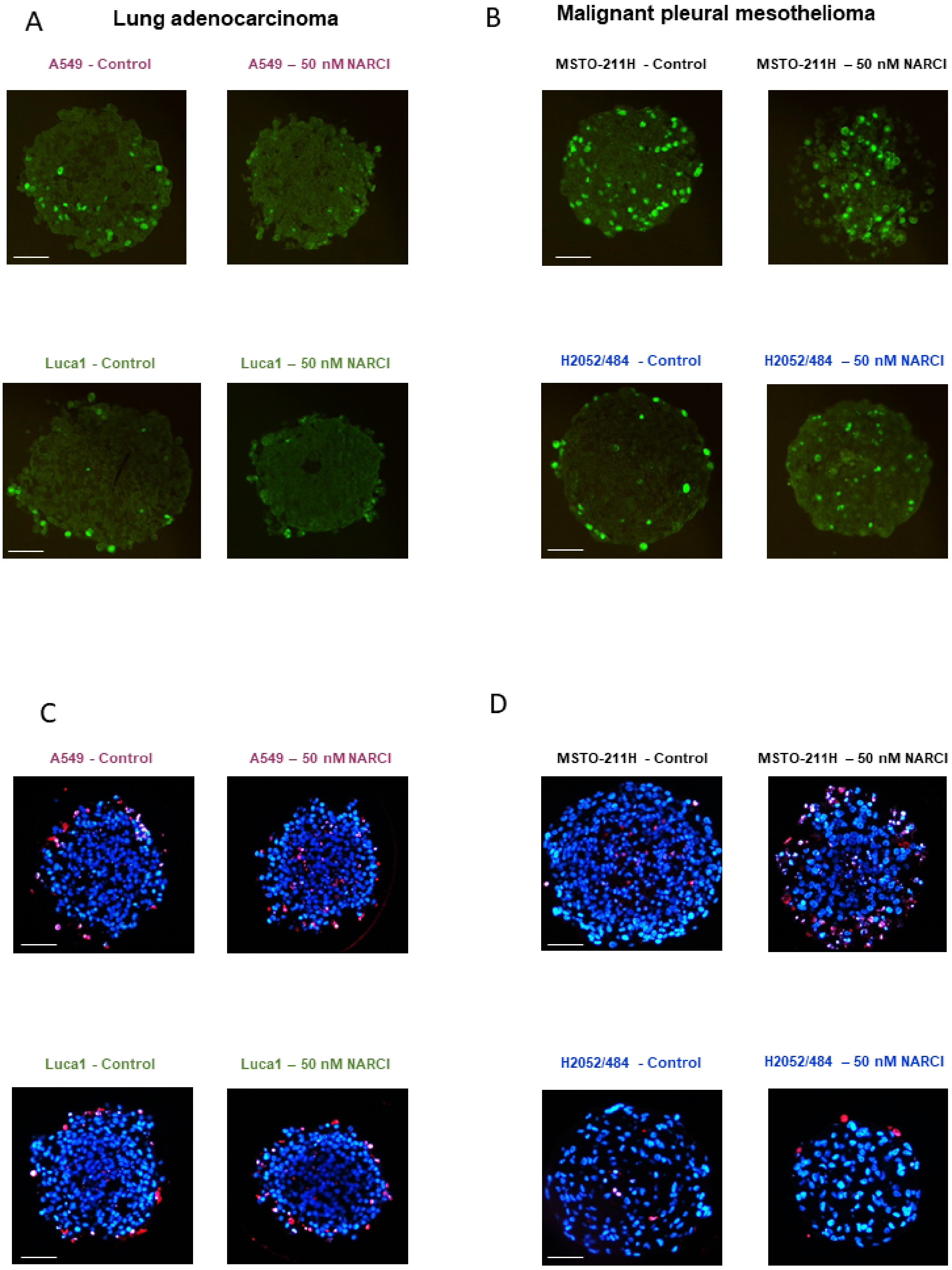

Figure S4. Narciclasine effect on the cuproptosis pathway in Lund adenocarcinoma.

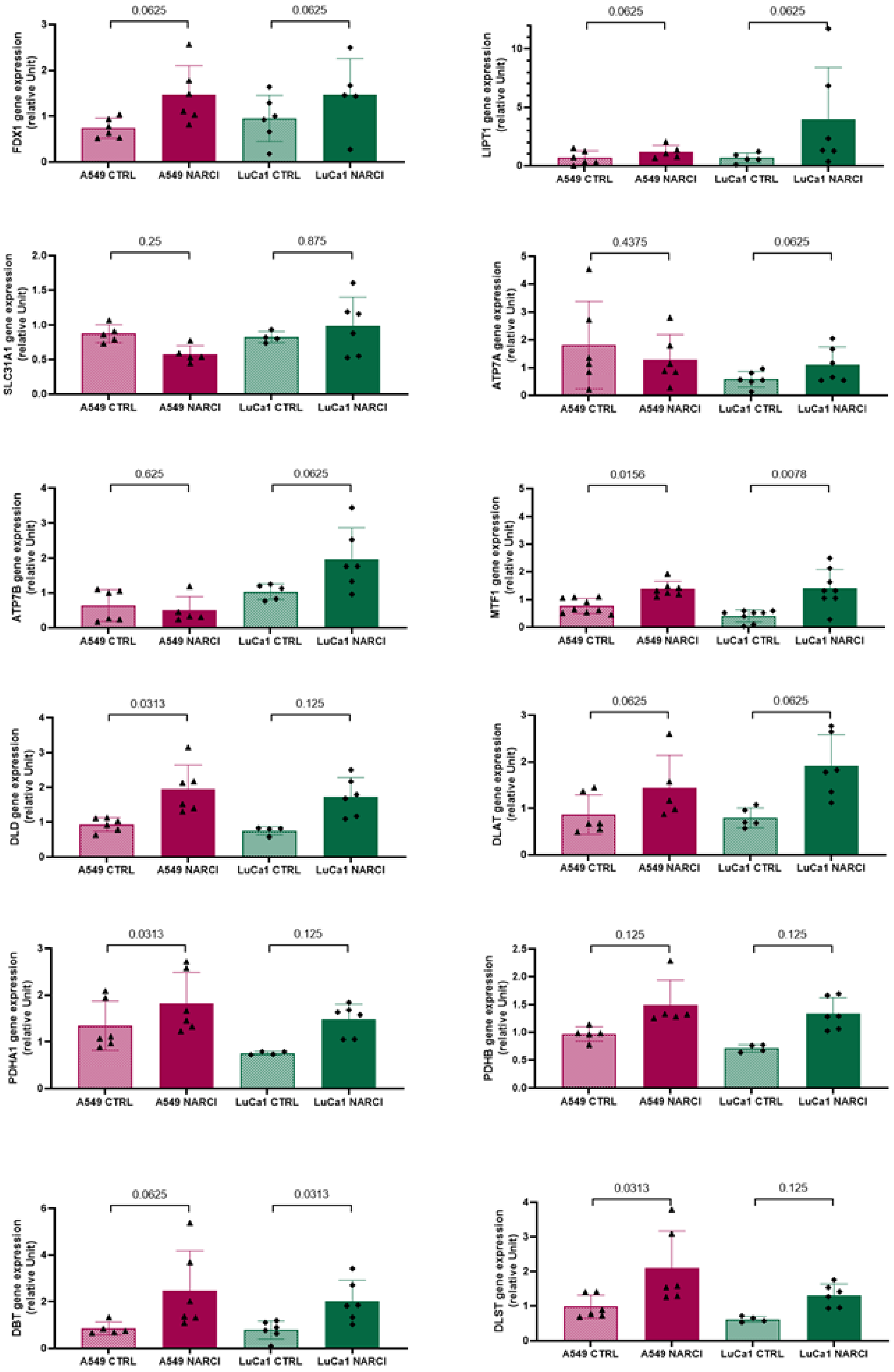

Figure S5. Narciclasine effect on the cuproptosis pathway in Pleural Mesothelioma.

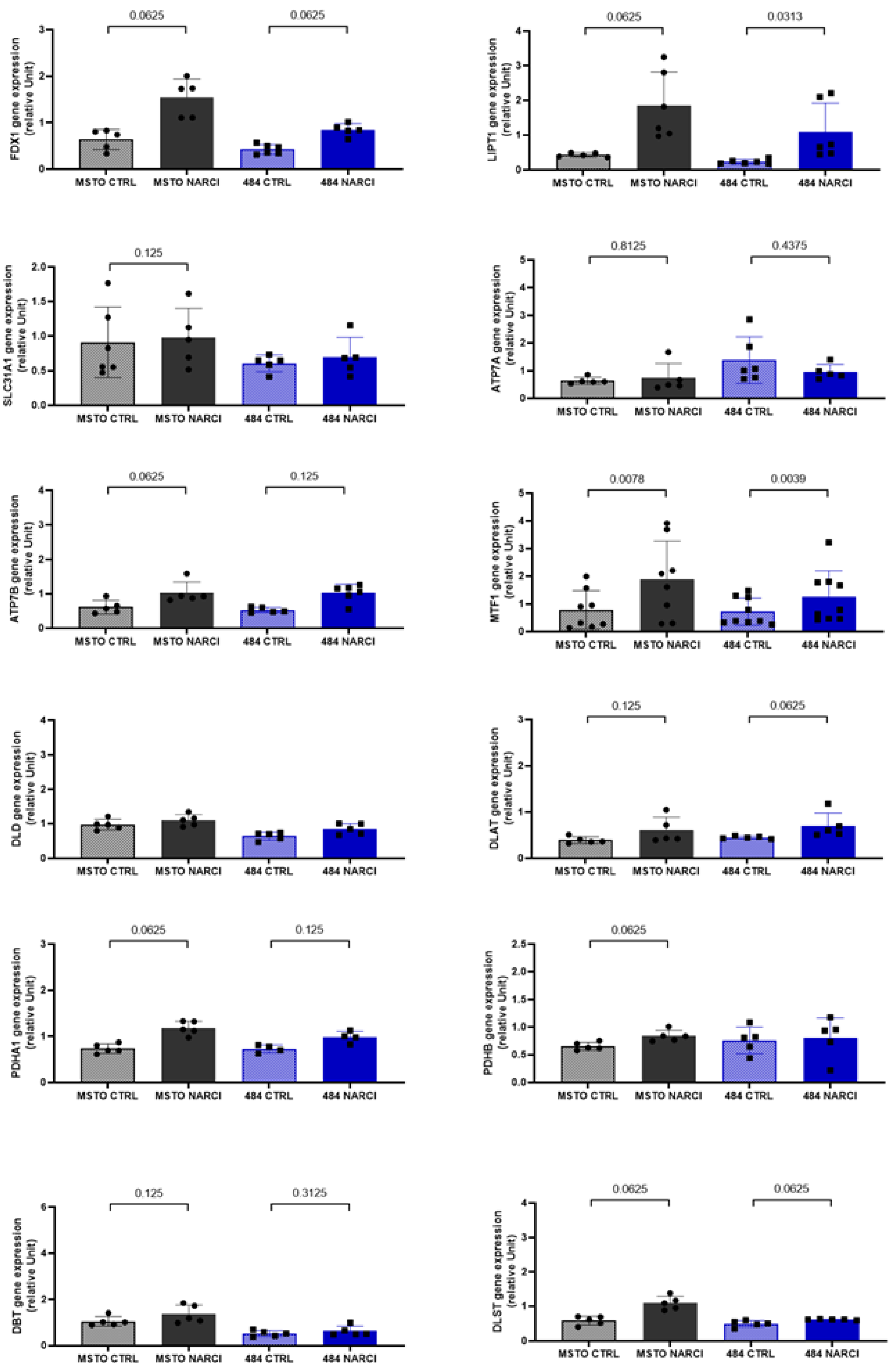

Figure S6. Narciclasine effect on the ferroptosis pathway in Lung adenocarcinoma.

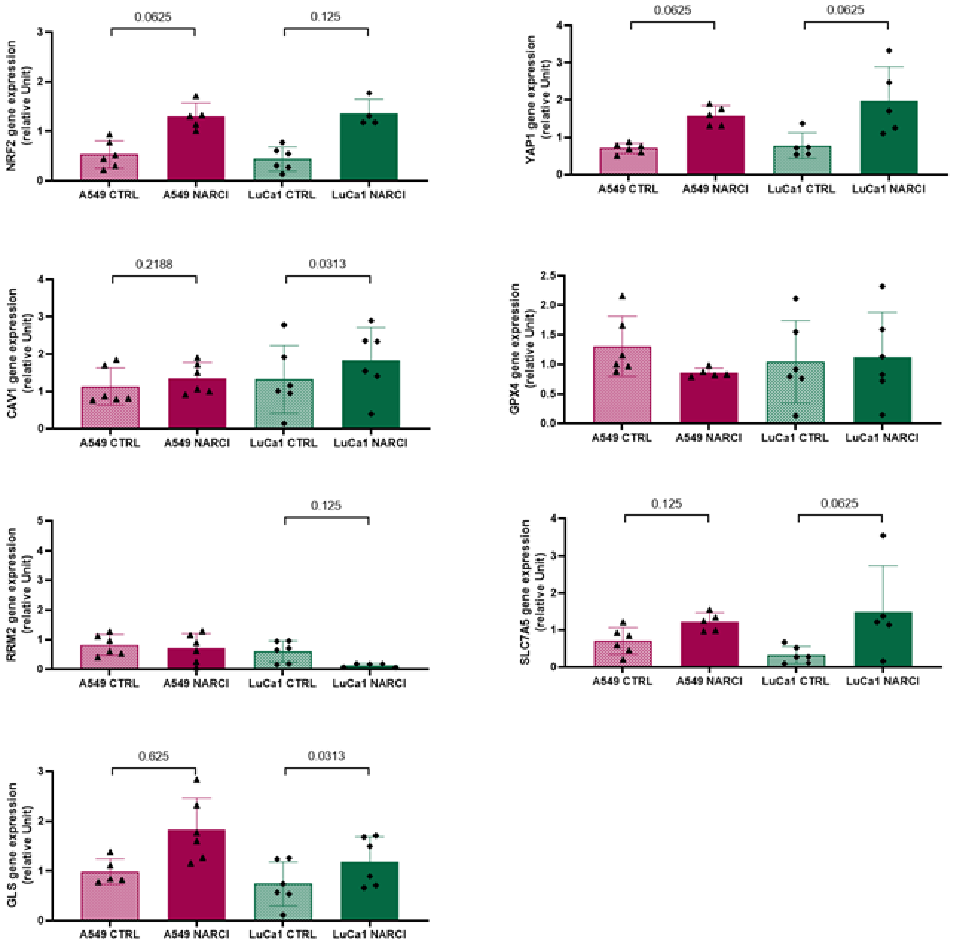

Figure S7. Narciclasine effect on the ferroptosis pathway in Pleural Mesothelioma.

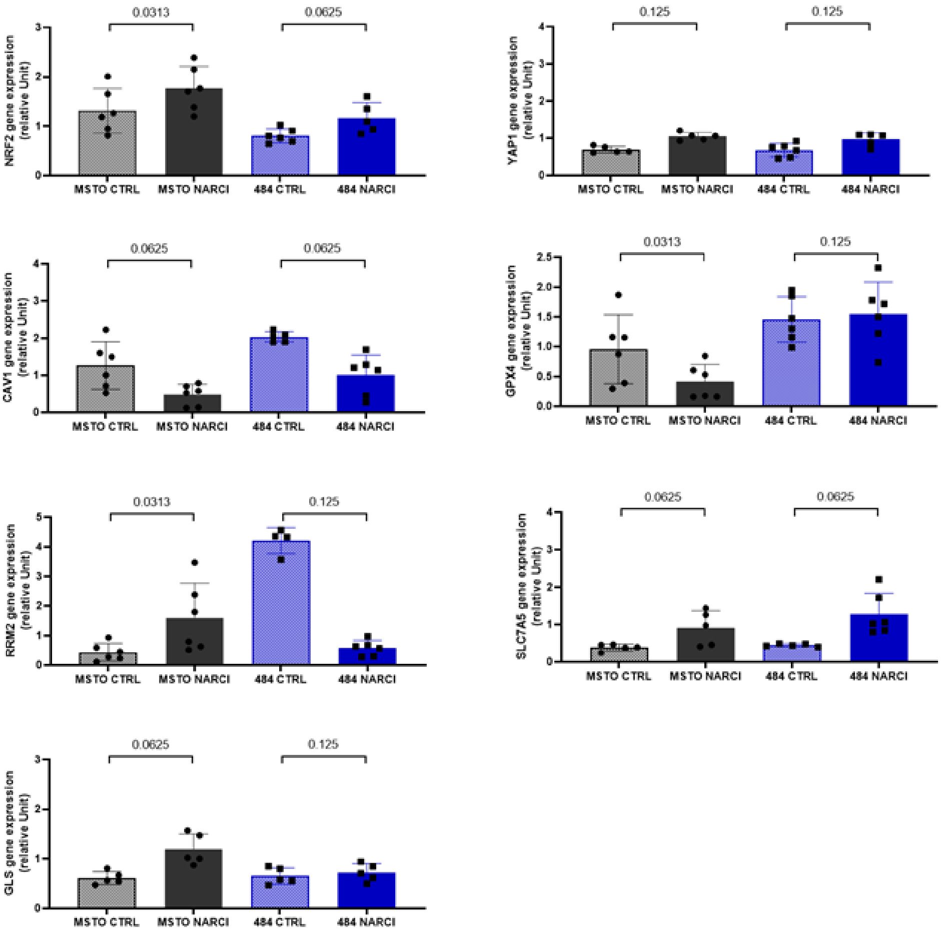

Figure S8. Narciclasine effect on the epithelial-to-mesenchymal transition in Lung adenocarcinoma.

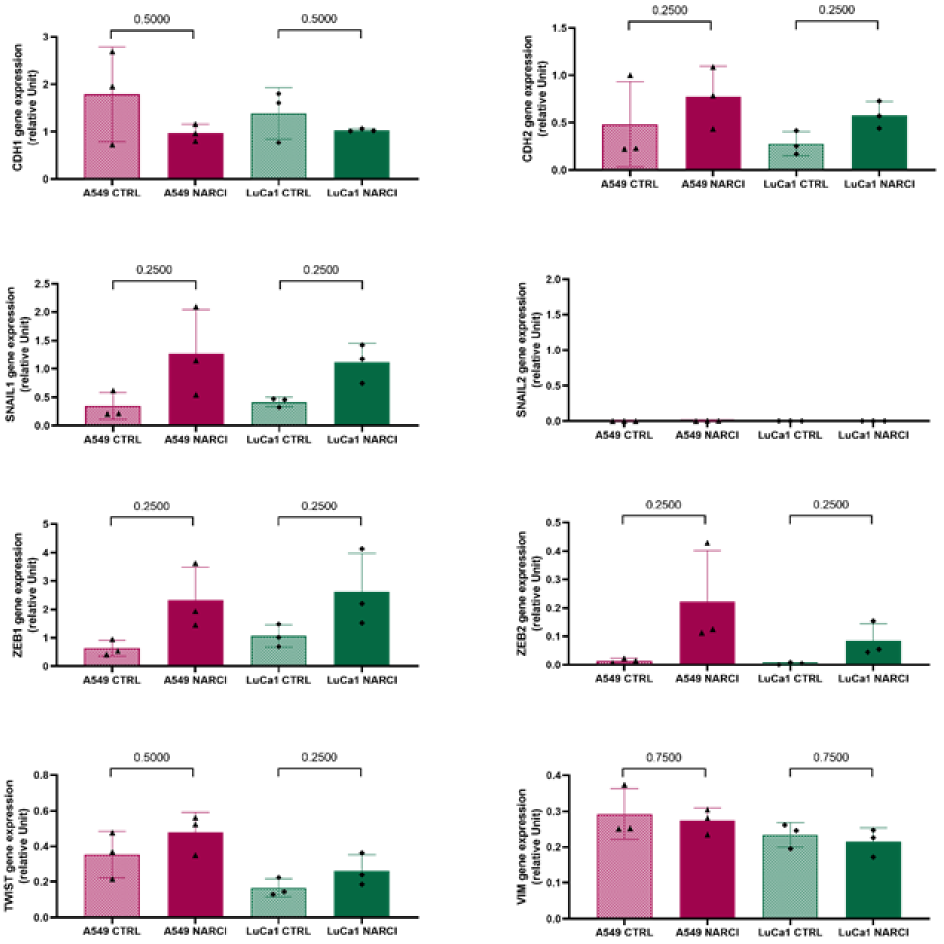

Figure S9. Narciclasine effect on the epithelial-to-mesenchymal transition in Pleural Mesothelioma.

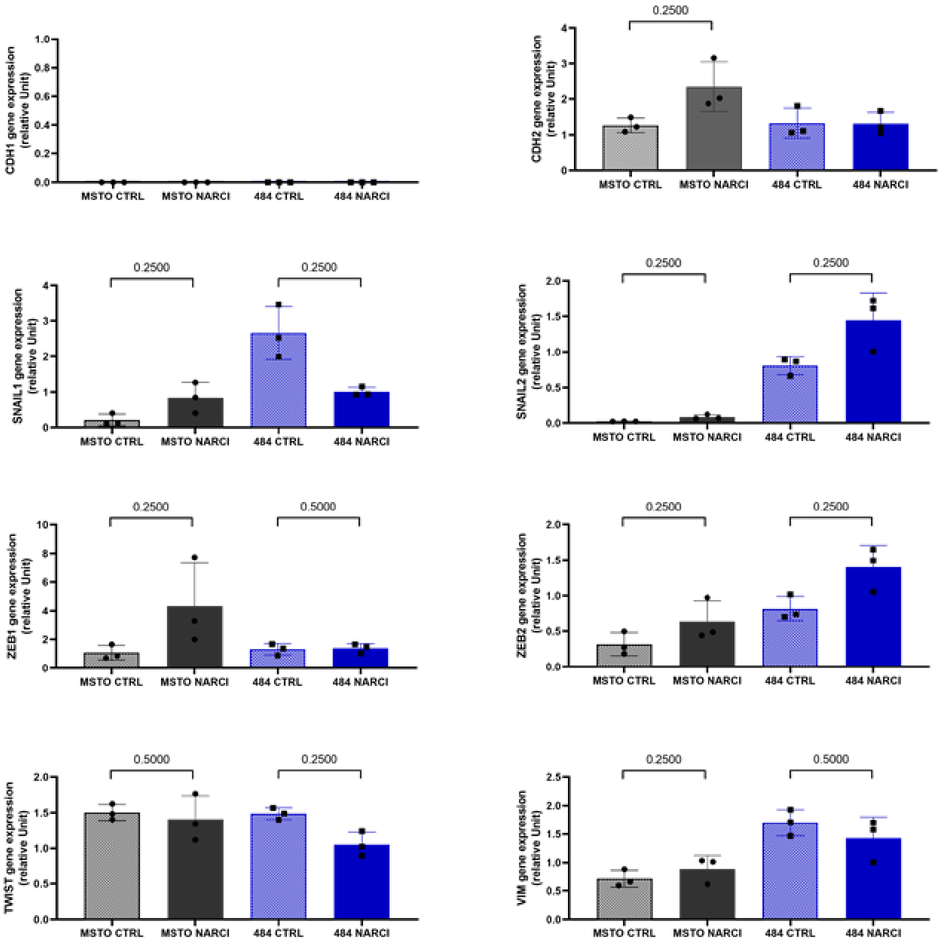

Supplement: Supplementary file 1 [file ijms-26-10127-s001.zip › ijms-3893708-supplementary.pdf]
